# ABL kinase inhibition sensitizes primary lung adenocarcinomas to chemotherapy by promoting tumor cell differentiation

**DOI:** 10.18632/oncotarget.26740

**Published:** 2019-03-08

**Authors:** Aaditya Khatri, Jing Jin Gu, Courtney M. McKernan, Xia Xu, Ann Marie Pendergast

**Affiliations:** ^1^ Department of Pharmacology and Cancer Biology, Duke University Medical Center, Durham, NC, USA

**Keywords:** lung adenocarcinoma, docetaxel, ABL kinases, differentiation, YAP1

## Abstract

Lung cancer is the leading cause of cancer mortality in the United States, with an overall five-year survival rate of ~16%. Non-small cell lung cancer (NSCLC) accounts for ~80% of all lung cancer cases, and the majority (40%) of these are adenocarcinomas. Loss of function point mutations in *TP53* (46%) and activating mutations in *KRAS* (33%) are the most common mutations in human lung adenocarcinomas. Because neither of these genetic alterations are clinically actionable, chemotherapy remains the mainstay of treatment in patients with oncogenic *KRAS* driver mutations. However, chemoresistance to genotoxic agents such as docetaxel remains a major clinical challenge facing lung cancer patients. Here we show that ABL kinase allosteric inhibitors can be effectively used for the treatment of *Kras^G12D/+^; p53^−/−^* lung adenocarcinomas in an autochthonous mouse model. Unexpectedly, we found that treatment of tumor-bearing mice with an ABL allosteric inhibitor promoted differentiation of lung adenocarcinomas from poorly differentiated tumors expressing basal cell markers to tumors expressing terminal differentiation markers *in vivo*, which rendered lung adenocarcinomas susceptible to chemotherapy. These findings uncover a novel therapeutic approach for the treatment of lung adenocarcinomas with poor response to chemotherapy.

## INTRODUCTION

With an estimated 234,000 new cases and 154,000 deaths in 2018, lung cancer is the leading cause of cancer deaths in the United States, accounting for one-quarter of all cancer deaths [1]. Approximately, 80% of lung cancer deaths are associated with smoking, which confers a 25-fold increase in relative risk. Smoking-associated lung cancer has one of the highest mutational burdens of all cancers [2]. Despite advancements in molecularly targeted therapies for patients harboring actionable genetic abnormalities such as mutations in *EGFR*, *ALK*, *RET*, or *BRAF*, the majority of lung cancers lack identifiable driver oncogenes or harbor mutations in *KRAS*, *TP53*, and other genetic abnormalities that are not actionable targets [2–4].

The *Kras^LSL-G12D^; p53^fl/fl^* mouse model was developed as a powerful model for studying lung adenocarcinomas [5, 6]. Tumor protein p53 (*TP53* or *p53*) (46%) and *Kirsten rat sarcoma viral oncogene homolog* (*KRAS*) (33%) are the most commonly mutated genes in human lung adenocarcinomas with a co-occurrence rate of ~15% [2, 7, 8]. Because neither genetic alteration is clinically actionable, chemotherapy remains the mainstay of treatment in patients with oncogenic *KRAS* driver mutations. Consequently, chemoresistance to genotoxic agents such as docetaxel and cisplatin remains an important clinical problem facing lung cancer patients.

The ABL kinases were initially identified as oncogenes in the context of patients with chronic myelogenous leukemia (CML) and acute lymphocytic leukemia (ALL) who presented with BCR-ABL1 fusion proteins due to a chromosomal translocation of *ABL1* to the *Breakpoint Cluster Region (BCR)* gene sequences [9]. Recent studies have identified a potential role for ABL kinases in solid tumors [10, 11]. Data from The Cancer Genome Atlas (TCGA) showed alterations of *ABL1* and *ABL2* in human lung adenocarcinomas, including copy number enhancement of *ABL2* and somatic mutations in *ABL1* in 1-2% of patients [2]. Further, *ABL2* genetic variations were identified in lung cancer in never smokers who were exposed to radon [12]. Importantly, increased ABL kinase activity has been detected in lung cancer cells without genomic alterations [13], and inactivation of ABL kinases suppresses lung cancer metastasis following intracardiac injection of NSCLC lines into immune-deficient mice [14].

Here, we demonstrate for the first time in the context of a *Kras^LSL-G12D^; p53^fl/fl^* (KP) mouse model of lung cancer that ABL kinase inhibition sensitizes primary lung adenocarcinomas to treatment with the chemotherapeutic agent, docetaxel. Further, we found that Abl inhibition promoted differentiation of the KP lung tumors, which was associated with increased cell death in the presence of docetaxel. Sensitization of lung tumors with Abl allosteric inhibitors to sub-therapeutic doses of chemotherapy would significantly decrease the deleterious side effects of chemotherapy and enhance response rates in patients with lung adenocarcinomas.

## RESULTS

### Inhibition of ABL kinases impairs *Kras^G12D/+^; p53^−/−^* driven lung tumors

**Figure 1:**
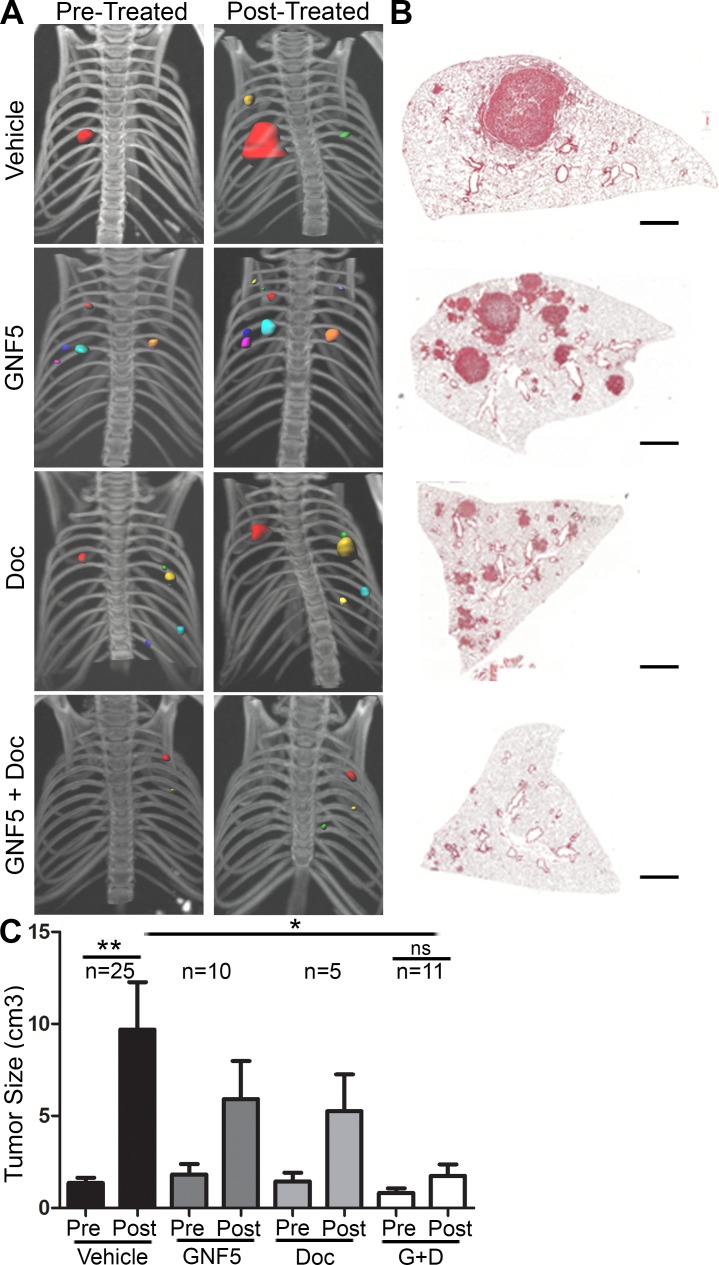
Inhibition of ABL kinases impairs Kras^G12D/+^; p53^−/−^ driven lung tumors Treatments began 8 weeks after Adeno-Cre infection of LSL-Kras^G12D/+^; p53^fl/fl^ mice. **A.** 3D-reconstructions of μ-CT scans of mice before and after 14 days of treatment with vehicle, GNF5 (100 mg/kg *b.i.d*.), docetaxel (10 mg/kg, twice weekly), or GNF5 (100 mg/kg) + docetaxel (10 mg/kg, twice weekly). **B.** H&E sections of lungs from mice in each treatment group 10 weeks after adenoviral induction. Scale bar = 2,000 μm. **C.** Quantification of total tumor volume in bilateral lungs evaluated by μ-CT scans taken before (8 weeks) and after (10 weeks) treatment with vehicle, GNF5 alone, docetaxel alone, or combination therapy shows that combination treatment significantly impairs tumor growth in mice compared to vehicle treated mice. Graphs depict means and S.E.M. of “n” mice, where “n” represents each individual animal used.

To evaluate whether ABL kinases play a role in the progression of primary lung adenocarcinomas, we evaluated whether pharmacological inhibition of the ABL kinases impaired tumor growth in an autochthonous *Kras^LSL-G12D^; p53^fl/fl^* (KP) mouse model [6]. Intranasal delivery of an adenovirus containing a Cre-recombinase expressing construct results in the activation of oncogenic *Kras* and loss of *p53* in infected cells. Consequently, spontaneous lung adenocarcinomas form throughout the lung approximately 8-12 weeks after viral delivery. Treatment with the Abl allosteric inhibitor, GNF5, and/or the chemotherapeutic agent, docetaxel, was initiated once tumor formation was confirmed by μCT scans performed at 8 weeks following infection (total tumor volume per mouse was < 1 cm^3^ with an average tumor diameter of < 3mm). GNF5 binds specifically to the myristoyl-binding site in the kinase domain of the ABL kinases, and is an Abl-specific inhibitor unlike the commonly used ATP-binding site inhibitors such as imatinib and nilotinib, which are known to interact with numerous other protein kinases [15]. We found that combination treatment with GNF5 and docetaxel markedly decreased tumor progression compared to vehicle control-treated mice or mice singly-treated with GNF5 or docetaxel alone (Figure 1A-1C).

### Combination treatment with Abl inhibitor and docetaxel decreases cell proliferation and increases cell death in *Kras^G12D/+^; p53^−/−^* driven lung tumors

**Figure 2:**
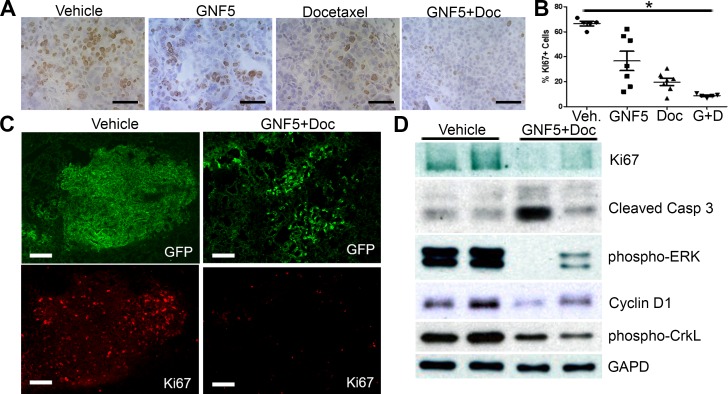
Inhibition of ABL kinases in the presence of docetaxel decreases cell proliferation and increases cell death in Kras^G12D/+^; p53^−/−^ driven lung tumors Treatments began 10 weeks after Adeno-Cre infection of *Rosa26-fGFP; LSL-Kras^G12D/+^; p53^fl/fl^* mice. Mouse lungs were harvested at 12 weeks after Adeno-Cre infection. **A.** IHC for Ki67+ cells in sections of mouse lungs from mice treated with vehicle control, docetaxel, GNF5, or combination (docetaxel+GNF5) treatment showing a decrease in Ki67 staining, particularly in the combination therapy group. Scale bar = 50 μm. **B.** Quantification of IHC staining for Ki67 (*n* = 5-7 tumors per group). **C.** Immunofluorescence staining for Ki67 showing a decrease in the percentage of Ki67+ (red) tumor cells (labeled with farnesylated GFP, green) in mice treated with combination therapy of GNF5 and docetaxel compared to vehicle treated mice. Scale bar = 150 μm. **D.** Immunoblotting of lysates showed a decrease in Ki67 expression and increase in cleaved caspase 3 expression in mice given combination therapy compared to control mice and a corresponding decrease in pERK and cyclin D1, which are downstream targets of oncogenic *Kras*. Phospho-CrkL is a marker for ABL kinase activity while GAPD is a loading control. Graphs depict means and S.E.M.

To evaluate the mechanism by which combination treatment with the Abl allosteric inhibitor, GNF5, and docetaxel inhibits lung adenocarcinoma growth *in vivo*, we harvested mouse lungs treated with vehicle control, GNF5 alone, docetaxel alone, or combination therapy two weeks after initiating treatment (12 weeks after delivery of adenovirus). Staining of lung tissue sections with the proliferation marker, Ki67, showed a profound decrease in Ki67 expression by immunohistochemistry (IHC) and immunofluorescence (IF) in double-treated mice compared to vehicle control mice or mice treated with GNF5 or docetaxel alone (Figure 2A-2C). We also found a significant decrease (~50% reduction) in expression of phospho-histone H3, a marker expressed specifically in mitotic cells, in mice treated with combination therapy compared to vehicle control treated mice (Supplementary Figure 1A and B).

Cell proliferation and apoptosis were evaluated in lung tumors derived from *Kras^LSL-G12D^; p53^fl/fl^* mice expressing the *Rosa26-fGFP* reporter, which allowed for isolation of GFP+ lung tumor cells by FACS analysis 12 weeks after delivery of adenovirus expressing Cre recombinase (Figure 2). Immunoblotting of protein lysates from the isolated GFP+ cells showed a marked decrease in Ki67 expression, as well as decreased levels of the Kras downstream targets cyclin D1 and phospho-ERK in mice treated with GNF5 and docetaxel (Figure 2D). To evaluate apoptosis, protein lysates from the isolated GFP+ cells were immunoblotted for cleaved caspase 3. We found an increase in expression of cleaved caspase 3 in a subset of GNF5 and docetaxel double-treated mice compared to vehicle control mice (Figure 2D). The increase in cleaved caspase 3 was primarily detected in double-treated tumors with greater inhibition of the cell proliferation markers phospho-Erk and cyclin D1 (Figure 2D). These data suggested that combination treatment with GNF5 and docetaxel inhibits cell proliferation and increases cell death in *Kras^G12D/+^; p53^−/−^* driven lung adenocarcinomas.

### Inhibition of ABL kinases sensitizes primary *Kras^G12D/+^* mouse-derived organoids and cell lines to treatment with docetaxel

**Figure 3:**
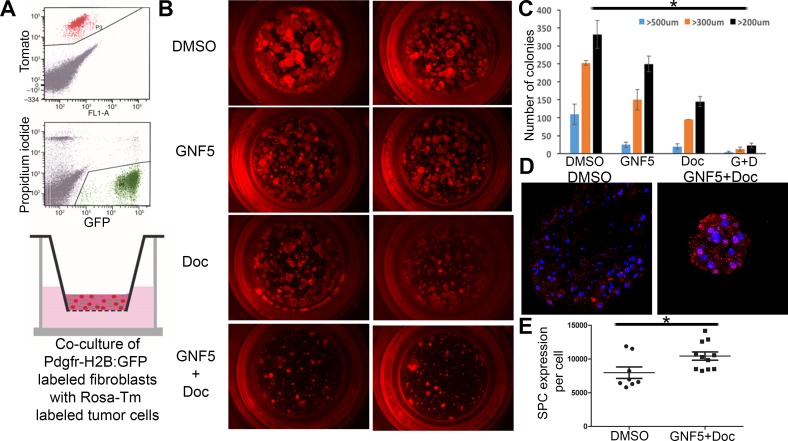
Inhibition of ABL kinases sensitizes primary Kras^G12D/+^ derived organoid cultures to treatment with docetaxel in 3D tumor sphere assays **A.** Model: *SPC (Sftpc)-CreERT2; KRAS^LSL-G12D^; Rosa26-tdTomato* mice were given tamoxifen to induce tumor formation. Tomato+ cells were then isolated from mice after tumor formation and grown in Matrigel in transwell inserts in the presence of primary mouse fibroblasts (derived from PDGFRα-H2B: GFP mice) to induce tumor organoid formation. **B and C.** Organoids were treated with vehicle (DMSO), GNF5, docetaxel (DOC), or combination treatment (GNF5 + DOC) for 2 weeks and assessed for organoid size 2 weeks after treatment (B); quantification revealed a significant reduction in organoid size in response to combination treatment compared to vehicle, GNF5, or docetaxel alone (C). Graphs depict means and S.E.M. **D.**-**E.** Immunofluorescence staining of organoids treated with GNF5 and docetaxel show increased expression of the Type II cell marker, SPC, compared to vehicle control treated mice. Quantification is provided in (E). Scale bar = 10 μm. Graphs depict means and S.E.M.

Next we evaluated the effects of ABL kinase inhibition on the growth of organoid cultures of primary tumors derived from *Kras^G12D/+^* mice. To this end, *SPC (Sftpc)-CreERT2; Kras^LSL-G12D^; Rosa26-tdTomato* mice were treated with tamoxifen to induce tumor formation specifically in Type II alveolar epithelial cells expressing the *SPC (Sftpc)* driver [16]. Tomato+ cells were isolated from mice after four weeks post-treatment and grown in Matrigel in the presence of primary mouse fibroblasts (GFP+) to assess tumor organoid formation (Figure 3A). Organoids were treated with vehicle control, GNF5 alone, docetaxel alone, or combination therapy starting three days after plating, and organoids were then grown for two weeks. We observed a decrease in the size of organoids treated with the combination therapy compared to vehicle- or single agent-treated organoids (Figure 3B-3C). By comparison, we observed no decrease in size or morphology of primary lung alveolospheres isolated from *SPC (Sftpc)-CreERT2; Rosa26-tdTomato* mice, expressing wild-type *Kras*, treated with GNF5 (Supplementary Figure 2A).

Several primary cell lines derived from *Kras^G12D/+^; p53^−/−^* mouse lung tumors were also evaluated in growth/viability assays using Cell-Titer-Glo in the presence or absence of the Abl allosteric inhibitor with and without docetaxel. We found significantly reduced cell growth in the combination treatment group compared to treatment with GNF5 or docetaxel alone (Supplementary Figure 3A). These data are consistent with *in vivo* data showing that ABL kinase inhibition sensitizes primary lung tumors to treatment with docetaxel (Figure 1).

### Inhibition of ABL kinases sensitizes *Kras^G12D/+^; p53^−/−^* lung adenocarcinomas to treatment with docetaxel by promoting cell differentiation

To further define the mechanisms by which ABL kinase inhibition sensitizes *Kras^G12D/+^; p53^−/−^* driven mouse lung tumors to docetaxel treatment, the expression of a variety of lung epithelial cell differentiation markers was evaluated following treatment with the ABL kinase inhibitor, GNF5, with and without docetaxel. A defining characteristic of adenocarcinomas is their progressive de-differentiation from low grade, glandular structures with well-differentiated tumor cells to high grade tumors that lack defined tissue morphology with poorly-differentiated tumor cells [17]. Thus, we hypothesized that ABL kinase inhibition may inhibit or reverse lung tumor de-differentiation.

**Figure 4:**
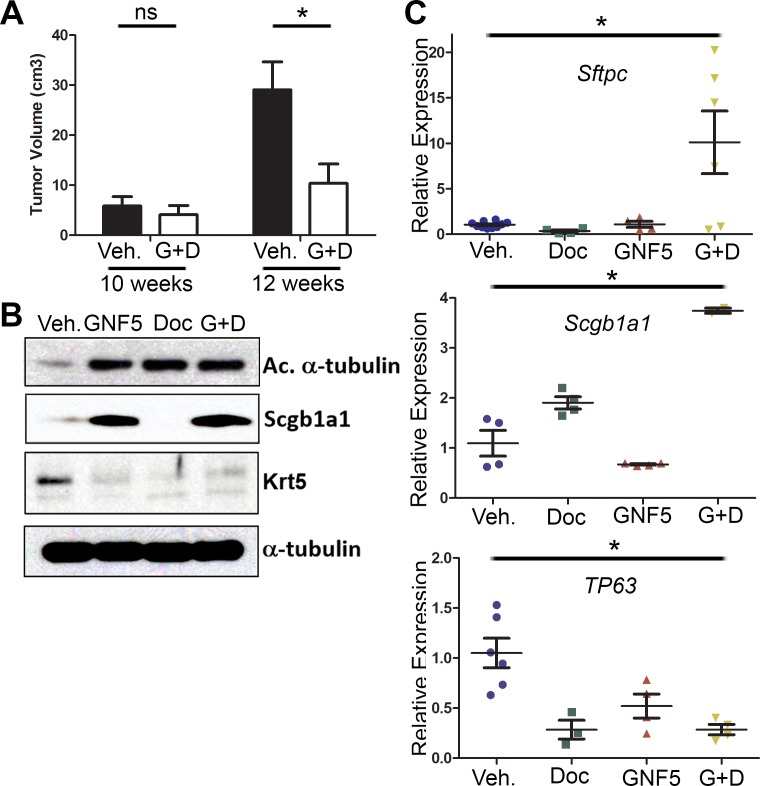
Combination treatment of GNF5 and docetaxel induces lung tumor cell differentiation *in vivo* GFP+ cells were isolated from *KRAS^LSL-G12D^; p53^fl/fl^; Rosa26-fGFP* mice two weeks after treatment with vehicle, docetaxel, GNF5, or combination treatment and 12 weeks after induction of tumors with adenovirus. **A.** Quantification of total tumor volume in bilateral lungs evaluated by μ-CT scans taken before (10 weeks) and after (12 weeks) treatment with vehicle or combination therapy (GNF5 + docetaxel) shows that combination treatment significantly impairs tumor growth in mice compared to vehicle treated mice (*n* = 11 mice per group). **B.** Western blot analysis showed an increase in expression of the ciliated cell marker, acetylated α-tubulin, and the secretory cell marker, Scgb1a1, with a corresponding decrease in expression of the basal cell marker, keratin 5, in mice treated with GNF5 and docetaxel. **C.** RT-qPCR analysis for indicated cell treatment groups shows an increase in expression of terminal cell markers (*Sftpc*: Type II cell marker and *Scgb1a1*: secretory cell marker) with a corresponding decrease in expression of the basal cell marker, *TP63*, in mice treated with the combination therapy compared to control or mice treated with GNF5 or docetaxel alone (*n* = 4-6 mice per group, each RT-PCR assay performed in triplicate). Graphs depict means and S.E.M.

We first evaluated whether combining Abl inhibition with chemotherapies altered the expression of differentiation marker genes *in vivo*. *Kras^LSL-G12D^; p53^fl/fl^* mice were crossed with mice expressing the *Rosa26-fGFP* reporter for isolation of GFP+ tumor cells by FACS. These mice were used for drug treatment following 10 weeks post-delivery of adenovirus-Cre (rather than 8 weeks after adenovirus-Cre as shown in Figure 1) in order to increase the size of the tumors in the combination therapy group and permit the isolation of sufficient quantities of protein for analysis of differentiation markers. This model is also more clinically relevant, as patients with lung adenocarcinomas typically present with moderate to poorly differentiated tumors [18]. Notably, μCT scans of mouse lungs before (10 weeks after viral induction) and after 2 weeks of drug treatment (12 weeks after viral induction) showed a three-fold reduction in tumor size for mice treated with both GNF5 and docetaxel compared to vehicle control treated mice (Figure 4A). Analysis of protein lysates from GFP+ tumors revealed an increase in terminal differentiation markers such as the ciliated marker, acetylated α-tubulin, and a corresponding decrease in the basal cell marker keratin 5 (Krt5) in mice treated with GNF5, docetaxel and combination therapy group compared to the vehicle control group (Figure 4B). Further, expression of the secretory cell marker CC10/Scgb1a1 was markedly enhanced by GNF5 and co-treatment of GNF5 + docetaxel (Figure 4B). Consistent with these results, RT-qPCR analysis of isolated GFP+ tumor cells isolated from mice co-treated with GNF5 and docetaxel showed a significant increase in expression of terminal differentiation markers (Type II cell marker, *SPC*, and secretory cell marker, *CC10*) with a concomitant decrease in expression of basal stem-cell markers including *p63* (Figure 4C). We also found a corresponding increase in SPC protein expression by IF analysis of mouse lung adenocarcinomas in response to the combination therapy of GNF5 with docetaxel (Supplementary Figure 4).

Consistent with these *in vivo* phenotypes, we found an increase in expression of terminally differentiated cell markers in two different *in vitro* assays in response to co-treatment with GNF5 and docetaxel. Analysis of tumor organoid cultures derived from *SPC (Sftpc)-CreERT2; Kras^LSL-G12D^; Rosa26-tdTomato* mice, showed that organoids from KRAS+ mice treated with the combination treatment of GNF5 and docetaxel had significantly higher expression of SPC compared to vehicle control-treated organoids from KRAS+ mice (Figure 3D-3E). Notably, vehicle control-treated organoids derived from KRAS+ mice demonstrated reduced expression of the Type II cell marker, SPC, compared to normal lung alveolospheres isolated from control KRAS-negative mice (Supplementary Figure 2B). Moreover, primary lung adenocarcinoma cell lines derived from *Kras^LSL-G12D^; p53^fl/fl^* lung tumors also showed an increase in expression of differentiation markers (acetylated α-tubulin) and a decrease in expression of basal cell markers (Krt5) after co-treatment with GNF5 and docetaxel compared to vehicle control or single-agent treatment *in vitro* (Supplementary Figure 3B). These *in vitro* data are consistent with the *in vivo* mouse data in showing that treatment with GNF5 and docetaxel promotes cell differentiation of lung adenocarcinomas.

### ABL kinases promote Yap1 signaling in Kras^G12D/+^; p53^−/−^ driven lung tumors

The Hippo pathway transcriptional regulatory factor Yap1 has been shown to regulate the proliferation and differentiation of lung epithelial cells during development and regeneration [19, 20]. Yap1 regulates organ size and morphology during development [21, 22]. Nuclear Yap1 is associated with pro-proliferative and anti-differentiation responses in lung airway cells and other tissues during development and tumorigenesis, and regulates expression of genes implicated in cell proliferation, including *c-Myc* [23–25]. In contrast, cytoplasmic Yap1 is associated with pro-differentiation and anti-proliferative responses [25]. Multiple cancer types are characterized by accumulation of nuclear Yap1, which is prevalent in highly proliferative, aggressive tumors and is associated with poor outcomes [26–28].

**Figure 5:**
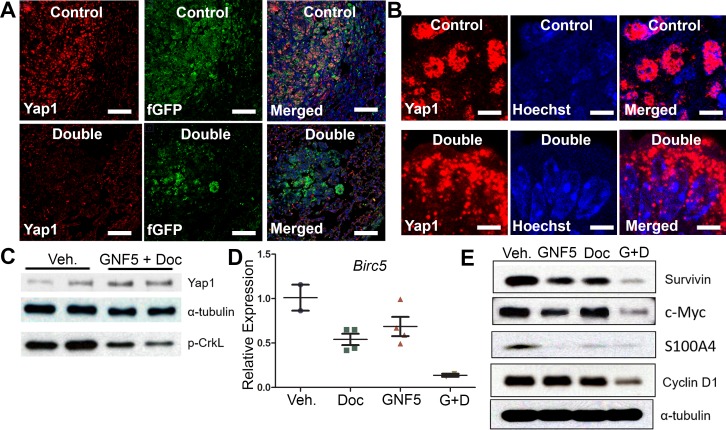
Combination treatment of GNF5 and docetaxel induces cytoplasmic localization of Yap1 and decreases expression of downstream transcription targets of Yap1 compared to vehicle control treated lung adenocarcinomas GFP+ cells were isolated from *KRAS^G12D/+^; p53^−/−^; Rosa26-fGFP* mice two weeks after treatment with vehicle, docetaxel, GNF5, or combination treatment and 12 weeks after induction of tumors with adenovirus. **A.** Immunofluorescence staining for Yap1 shows a decrease in Yap1 nuclear localization in mice treated with GNF5 and docetaxel compared to vehicle control treated mice. Scale = 75 μm. **B.** Higher magnification images are provided to show sub-cellular localization of Yap1 in control and combination treatment mice. Scale = 7.5 μm. **C.** Western blot analysis showed no significant difference in total Yap1 protein expression in double treated mice compared to vehicle treated control mice. Phospho-CrkL expression is a marker of ABL kinase activity. **D.** RT-qPCR analysis showed a decrease in mRNA transcript expression of the downstream Yap1 target, *Birc5*, which encodes the protein survivin, in mice treated with both GNF5 and docetaxel compared to vehicle control treated mice or mice treated with GNF5 or docetaxel alone (*n* = 3-4 mice per group, each RT-qPCR assay performed in triplicate). **E.** Western blot analysis showed a decrease in protein expression of downstream transcriptional targets of Yap1, including survivin, c-Myc, S100A4, and cyclin D1. Graphs depict means and S.E.M.

We evaluated Yap1 expression in *Kras^G12D/+^; p53^−/−^* driven mouse lung tumors by immuno-staining of lung tissue sections from mice treated with vehicle control or the combination therapy of GNF5 and docetaxel. High-level expression of nuclear Yap1 in vehicle control treated mice was markedly reduced in mice treated with the combination therapy, which promoted a punctate pattern of Yap1 cytosolic localization (Figure 5A-5B). Notably, total Yap1 protein expression was unchanged in vehicle control treated mice compared to mice in the combination therapy group (Figure 5C).

To determine whether the decrease in nuclear Yap1 staining resulted in decreased expression of Yap1 transcriptional targets, we isolated protein lysates from GFP+ lung adenocarcinoma cells derived from *Kras^G12D/+^; p53^−/−^; Rosa26-fGFP* mice treated with vehicle control, GNF5 alone, docetaxel alone, or combination therapy and lysates were analyzed by Western blotting for known Yap1-regulated targets. We found a profound decrease in the expression of Yap1 targets including Survivin, c-Myc, S100A4, and Cyclin D1 in mice treated with GNF5 and docetaxel (Figure 5E). We also detected a corresponding decrease in *Survivin*/*Birc5* mRNA transcript levels in lung tumors isolated from *Kras^G12D/+^; p53^−/−^; Rosa26-fGFP* mice treated with combination therapy (Figure 5D). These data suggest that combination treatment with an Abl allosteric inhibitor and docetaxel inhibits nuclear Yap1 signaling in these tumors.

## DISCUSSION

Chemotherapy is a first-line treatment in the majority of patients with lung adenocarcinomas that often fails due to intrinsic or acquired resistance. Thus, combination therapies that sensitize tumors to chemotherapy might have a profound impact in the treatment of these patients. Here we show that ABL kinase inhibition sensitizes *Kras^G12D+/−^; p53^−/−^* tumors to treatment with docetaxel such that sub-therapeutic doses of docetaxel confer reduced cell growth and increased cell death both *in vivo* and *in vitro* in the presence of Abl allosteric inhibitors.

Tumors frequently co-opt pathways implicated in development and regeneration to promote cell growth and inhibit cell death. As adenocarcinomas progress from less aggressive, well-differentiated tumors with glandular morphology reflecting their cell of origin (low grade tumors) to aggressive, poorly-differentiated tumors that lack any identifiable morphology (high grade tumors), they undergo a de-differentiation process that includes reduced expression of markers characteristic of their cell of origin and increased expression of basal cell markers or other ectopic protein expression markers [29]. Some lung tumors lose their epithelial morphology through an epithelial-mesenchymal transition (EMT). We have previously shown that ABL kinases are upregulated in metastatic lung tumors and promote differentiation of lung epithelial cells in the context of bacterial pneumonia [30]. We hypothesized that inhibition of the ABL kinases might promote differentiation of lung adenocarcinomas from more de-differentiated, basal-like tumors to differentiated tumors expressing terminal cell differentiation markers such as SPC. The data presented here support this hypothesis using an autochthonous *Kras^G12D/+^; p53^−/−^* lung cancer mouse model, lung cancer organoid 3D cultures and primary 2D cell cultures. We found increased expression of pro-differentiation markers and decreased expression of basal cell markers in lung adenocarcinoma-bearing mice or lung tumor cells treated with the ABL kinase inhibitor, GNF5, compared to vehicle control treated mice.

ABL kinase inhibition may drive differentiation of lung cancer cells through modulation of the Hippo pathway. Nuclear Yap1 promotes proliferative pathways in cells and translocation of Yap1 to the cytoplasm is associated with a switch from proliferative to pro-differentiation signaling [21, 31]. Accumulating data suport roles for Yap1 in tumor progression and chemoresistance [32, 33]. *KRAS*-mutated human non-small cell lung cancers are associated with increased nuclear YAP1, and downregulation of YAP1 has been shown to synergize with the chemotherapeutic agent, cisplatin [34]. Nuclear YAP1 was shown to promote cancer cell survival by activating Survivin expression [35]. Several distinct mechanisms have been reported to regulate YAP1-mediated regulation of therapy resistance in lung cancer cells including acquistion of an EMT phenotype and overexpression and/or activating mutations of receptor tyrosine kinases [36]. Activation of Yap1 and various other oncogenic pathways converge to activate MYC and drive chemoresistance [37]. Inhibition of ABL kinases would blunt signaling to Yap1 and other factors linked to chemoresistance, and might be exploited as a potential strategy to treat lung tumors. Because of the high toxicity profiles of compounds that target Yap1 [38], the use of Abl allosteric inhibitors may be a promising new therapeutic strategy for lung adenocarcinoma patients.

## MATERIALS AND METHODS

### Mouse experiments

*Kras^LSL-G12D^; p53^fl/fl^* mice were kindly provided by Dr. David Kirsch at Duke University. To induce lung tumors, mice were given 30 μL virus dilution in DMEM followed by 30 μL of DMEM intranasally in 6-week-old mice under isofluorane anesthesia. The virus dilution was prepared by diluting 44 μL viral stock (VVC U of Iowa-5; University of Iowa Vector Core Facility) in 500 μL DMEM with 2.5 μL 2M calcium chloride. Mouse tumors were evaluated by conducting complete body μCT scans 8 or 10 weeks after viral induction for tumor inhibition and tumor regression studies, respectively. In mice in which tumors were identified, treatment with GNF5 (100 mg/mL *b.i.d. via* oral gavage), docetaxel (10 mg/kg twice/week as indicated *via* intraperitoneal injection), or combination treatment was initiated for two weeks. After two weeks, follow-up complete body μCT scans were performed to evaluate change in tumor size. Analysis of tumor size was then performed using Imaris Version 8 software.

### Drug preparation

GNF5 (N-(2-Hydroxyethyl)-3-(6-(4-(trifluoromethoxy)phenylamino) pyrimidin-4-yl) benzamide) was synthesized at the Duke University's Small Molecule Synthesis Facility, and validated by LC-MS and 1H-NMR/FT-IR spectra and with cell-based assays that confirm potencies and cell signaling inhibitory activities. For *in vivo* experiments, GNF5 was prepared in a suspension with 0.5% methylcellulose and 0.5% Tween-80 at a concentration of 10 mg/mL, and mice were treated with 100 mg/kg *b.i.d. via* oral gavage.

Docetaxel (LC Laboratories: D-1000) stock solution was prepared by dissolving the powder at a concentration of 30 mg/mL in ethanol. Working dilutions were prepared fresh each day by diluting the stock solution 1:10 in 5% sucrose water containing 10% Tween 80. The working dilutions were filtered using 0.4 μm filters and given to mice intra-peritoneally at a dose of 10 mg/kg twice weekly.

### Preparation of lung tissue sections

Mice were euthanized after two weeks of treatment either 10 or 12 weeks after viral induction. The aorta was dissected to reduce intravascular blood volume, and the lungs were exposed through an incision across the diaphragm and the bottom half of the sternum. The trachea was exposed and cannulated with a sterile 22-gauge Abbocath-T catheter to inflation fix the mouse lungs. Fixation with 4% paraformaldehyde solution in PBS was performed for 15 minutes at room temperature by suspending fixation solution in an apparatus 40 cm above the mice. The entire mediastinum including the trachea, lungs, and heart were then carefully excised and immediately placed in 4% paraformaldehyde for 4 hours on a rotator at 4°C. Specimens were then washed three times for 15 minutes each with PBS. The left lungs were transferred to 70% ethanol overnight, paraffin embedded at the Duke University Immunohistopathology Core Facility, and cut to 5 μm thick sections. The right lungs were sucrose protected (30% sucrose in water), frozen in Optimal Cutting Temperature (OCT) compound at −80°C, and cut to 5 μm thick sections.

### Hematoxylin and eosin staining and quantification

Tissue sections were deparaffinized, rehydrated, and incubated with hematoxylin staining reagent for 10 minutes followed by treatment of acid alcohol, Scott's water, and an eosin secondary counter-stain for 1 minute each. Slides were cleared by xylene, mounted with mounting medium, and analyzed on a Zeiss Axio Imager upright microscope using a 10x objective. Entire left lung coronal sections at approximately the same depth were obtained for each mouse of each experimental condition. Lung sections were imaged and stitched together using the Zeiss Zen software to produce whole left lung images. The fraction of tumor area was determined using the particle analysis function on FIJI. A one-way ANOVA followed by a post-hoc Tukey test was performed to evaluate the differences in tumor area between each group. No image adjustments were applied to H&E images prior to quantification.

### Immunofluorescence

Left lung tissue sections were deparaffinized, rehydrated, and heat inactivated (BioCare Medical Decloaking Chamber). Right lung tissue sections were thawed at room temperature for 15 minutes. Both deparaffinized and frozen sections were then washed in PBS and blocked in 3% goat serum in PBS with 0.05% Tween-20 for one hour. Sections were incubated with primary antibodies in blocking solution overnight at 4°C in a humidified chamber at concentrations indicated below. Sections were then washed with PBS followed by incubation with the appropriate secondary antibody in blocking solution for one hour at room temperature. Sections were then washed with PBS, incubated with the nuclear stain, Hoechst33342, and washed again with PBS before mounting using aqueous mounting media (Dako-S3025). Antibodies for immunofluorescence and IHC experiments included: Anti-SPC (Millipore-AB3786) at a 1:1000 dilution, anti-GFP (Aves Labs-GFP-1020) at a 1:1000 dilution, anti-pHH3 (Sigma-Aldrich-H9908) at a 1:1000 dilution, and anti-Yap1 (Abnova-H00010413-M01) at 1:250 dilution. Secondary antibodies with fluorescent labels were purchased from ThermoFisher (anti-mouse, anti-rabbit, and anti-rat Alexa Fluors 488, 561, and 647) and used at 1:1000 dilutions.

### Quantification of immunofluorescence experiments

All cell counts were evaluated for entire left lung sections using a 10x objective on the Zeiss AxioImager microscope using Zen software at the Duke Light Microscope Core Facility. Cell counts were validated, and representative high-resolution images were taken using 40x and 100x objectives on the Leica SP8 confocal microscope. Stitched images were quantified using the Analyze Particle feature in Fiji software to determine total cell counts across the entire lung for each mouse. No image adjustments were applied to 10x objective stitched images prior to quantification. Representative high-resolution images displayed in the figures were modified along a linear scale for ease of viewing due to differences in sensitivities of antibodies.

### FACS

Mice were euthanized after two weeks of treatment either 10 or 12 weeks after viral induction. The aorta was dissected to reduce intravascular blood volume, and the lungs were exposed through an incision across the diaphragm and the bottom half of the sternum. The trachea was exposed and cannulated with a sterile 22-gauge Abbocath-T catheter to inflation fix the mouse lungs with enzyme solution containing 4.5 mg Type I collagenase (ThermoFisher: 17100017), 100 μL elastase (4U/mL) (Worthington Biochemical Corporation: LS002279), 300 μL dispase (5U/mL) (BD Biosciences: 354235), and 200 μL Dnase I (0.33U/mL) (Roche: 10104159001). Lungs were dissociated by enzyme digestion by first separating them from surrounding tissue, cutting into small pieces using scissors, and incubating at 37°C in the enzyme solution with vigorous shaking every five minutes. Once homogenized, 8mL DMEM-F12 solution containing 10% FBS was added to neutralize the enzyme solution. The cell suspension was then filtered through a 70 μm strainer, centrifuged, and resuspended in red blood cell lysis buffer (ThermoFisher: 00-4333-57). 8mL DMEM-F12 solution containing 10% FBS was added to neutralize the lysis buffer. The cell suspension was then centrifuged, resuspended in 1mL DMEM-F12 containing 2% BSA, and filtered using a 40 μm cell strainer for FACS. FACS was performed using a BD-DiVa system at the Duke Flow Cytometry Core Facility. RNA or protein was collected from isolated GFP-labeled cells using an RNA isolation kit (GE-25050071) or RIPA buffer digestion (containing protease and phosphatase inhibitors), respectively.

### RT-PCR

RNA was isolated from cells using an RNA isolation kit (GE-25050071), and complementary DNA was synthesized using oligo(dT) primers and Moloney murine leukemia virus reverse transcriptase (Invitrogen). Real-time PCR was performed using iQ SYBR Green Supermix (BioRad-1708882). The primers used were as follows: mouse *Scgb1a1*, ATCGCCATCACAATCACTACTG (forward) and CAGTCTCTTCAGCTGGGTGC (reverse); mouse *Gapdh*, AGGTCGGTGTGAACGGATTTG (forward) and TGTAGACCATGTAGTTGAGGTCA (reverse); mouse *Sftpc*, AACGCCTTCTCATCGTGGT (forward) and TAGATATAGTAGAGTGGTAGCT (reverse); mouse *Birc5*, AAGGAATTGGAAGGCTGGG (forward) and TTCTTGACAGTGAGGAAGGC (reverse); mouse *TP63*, CCCACAGACTGCAGCATTG (forward) and GAGATGAGGAGGTGAGGAGAAG (reverse). Analysis was performed using a BioRad CFX384 real-time machine and CFX Manager software. PCR assays were performed in duplicate. The expression of each gene was normalized to that of *Gapdh* (mouse).

### Immunoblotting

Cells were lysed in radioimmunoprecipitation assay (RIPA) buffer (50mM Tris-HCl, pH 7.5, 150mM NaCl, 1% Triton X-100, 0.1% SDS, and 0.5% sodium deoxycholate with protease and phosphatase inhibitors. Cell debris was removed by microcentrifugation, and protein concentration was quantified using the DCTM Protein Assay (Bio-Rad Laboratories). Equal amounts of protein were separated by SDS-polyacrylamide gel electrophoresis (BioRad-1610183) and transferred onto 0.2 μm pore nitrocellulose membranes. Membranes were probed with the appropriate antibodies overnight at 4°C at dilutions indicated below, thoroughly washed, and then incubated with secondary HRP-tagged antibodies (Jackson Laboratory) for one hour at room temperature. Blots were incubated using chemiluminescent reagents (ThermoFisher-34580) and developed using x-ray film (GE-45001).

**Table:** 

Antibody	Source	Catalog number
Ki67	BD pharmingen	550609
c-Myc	Cell Signaling	5605
Cleaved Caspase 3	Cell Signaling	9661
p-CrkL (y207)	Cell Signaling	3181
Cyclin D1	Cell Signaling	2978
S100A4	abcam	Ab27957
Survivin	Cell Signaling	2808
p-Erk (T202/Y204)	Cell Signaling	4376
Acetyl-α-tubulin	Cell Signaling	5335
Keratin 5	BioLegend	905501
Scgb1a1 (CC10)	Millopore	07-623
α-tubulin	Millipore-Sigma	05-829

### Tumor spheroid assay

6–8 weeks old *Kras^LSL-G12D/+^; Sftpc-CreERT2; LSL-TdTomato* mice were administrated with Tamoxifen (T5648; Sigma-Aldrich) at 20 mg/ml stock solution in corn oil and given *via* intraperitoneal injection of 0.25 mg/g. 4 weeks after Tamoxifen injection, *Kras^LSL-G12D/+^; Sftpc-CreERT2; LSL-TdTomato* and *PDGFRα-eGFP* mouse lungs [39] were dissociated using enzymatic digestion as described above. TdTomato positive cells and GFP positive cells were sorted using a BD-DiVa system at the Duke Flow Cytometry Core Facility. Viable Tdtomato single positive cells (5,000 cells) were mixed with PDGFRα-GFP positive fibroblasts (100,000 cells) in 1:1 Matrigel/MTEC plus media for a total volume of 100 μL in 24-well (0.4-μm) Transwell filter inserts. 500 μL MTEC plus medium was placed in the lower chamber and changed every other day. Cultures were treated with GNF5 (5 μM) and/or docetaxel (1nM) and maintained in a humidified 37°C incubator in 5% CO_2_. After two weeks, tumor sphere sizes were evaluated for each group.

### Viability and proliferation assay

*Kras^G12D/+^; p53^−/−^* mouse cell lines were graciously provided by Kwok-Kin Wong (New York University) and Jonathan Kurie (MD Anderson). Cells (2×10^3^) were seeded in 96 well plates in triplicates, drugs were added at the indicated concentrations the following day and measured each day using Celltiter-glo (Promega).

### Statistical analysis

The number of animals in each group is indicated in the figures and/or figure legends. Differences among groups for immunofluorescence and H&E experiments were assessed using one-way ANOVA followed by post-hoc Tukey tests. All quantification for these experiments was performed using stitched images for the entire left lung (> 100,000 cells counted per image). Representative high-resolution images from each animal group are indicated in the figures. Results are presented as means +/− standard error of measurement. Individual group testing was performed only when statistical significance was achieved from the ANOVA. Real-time RT-qPCR experiments were all performed in triplicate in three separate mouse samples. “*”, “**”, and “***” indicate *p*-values, < 0.05, < 0.01, and < 0.001, respectively. Bar graphs represent means with standard errors of measurement.

## SUPPLEMENTARY MATERIALS FIGURES



## Abbreviations

ANOVA: analysis of variance
CC10: Clara cell 10 protein
FACS: fluorescent activated cell sorting
GFP: green fluorescent protein
H&E: Hemotoxylin & Eosin
HRP: horse radish peroxidase
μCT: X-ray microtomography
PDGFR: platelet derived growth factor receptor
RIPA: radioimmunoprecipitation assay
RT-qPCR: Real time quantitative polymerase chain reaction
SPC: surfactant protein C
