# Correction: ABL kinase inhibition sensitizes primary lung adenocarcinomas to chemotherapy by promoting tumor cell differentiation

**DOI:** 10.18632/oncotarget.27258

**Published:** 2019-10-15

**Authors:** Aaditya Khatri, Jing Jin Gu, Courtney M. McKernan, Xia Xu, Ann Marie Pendergast

**Affiliations:** ^1^ Department of Pharmacology and Cancer Biology, Duke University Medical Center, Durham, NC, USA

**This article has been corrected: **In Figure 2D, the panel for the loading control should be labeled ‘tubulin’, not ‘GAPD.’ We have corrected the legend to Figure 2D by updating this sentence: “Phospho-CrkL is a marker for Abl kinase activity while α-tubulin is a loading control.” In addition, we have added a sentence to the legend of Figure 5C to clarify the origin of the lysates: “C) Western blot (using the same lysates as Figure 2D) analysis showed no significant difference in total Yap1 protein expression in double treated mice compared to vehicle treated control mice.” The corrected Figure 2D and the corrected legend of Figure 5 are shown below. The authors declare that these corrections do not change the results or conclusions of this paper.

Original article: Oncotarget. 2019; 10:1874–1886. 1874-1886. https://doi.org/10.18632/oncotarget.26740

**Figure 2:**
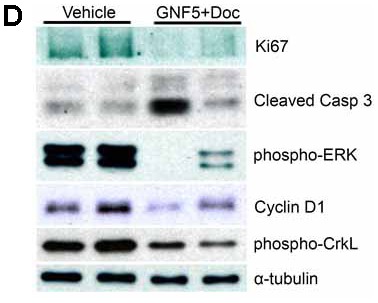
Inhibition of Abl kinases in the presence of docetaxel decreases cell proliferation and increases cell death in Kras^G12D/+^; p53^-/-^ driven lung tumors. (**D**) Immunoblotting of lysates showed a decrease in Ki67 expression and increase in cleaved caspase 3 expression in mice given combination therapy compared to control mice and a corresponding decrease in phospho-ERK and cyclin D1, which are downstream targets of oncogenic *Kras*. Phospho-CrkL is a marker for Abl kinase activity while α-tubulin is a loading control. Graphs depict means and S.E.M.

**Figure 5:**
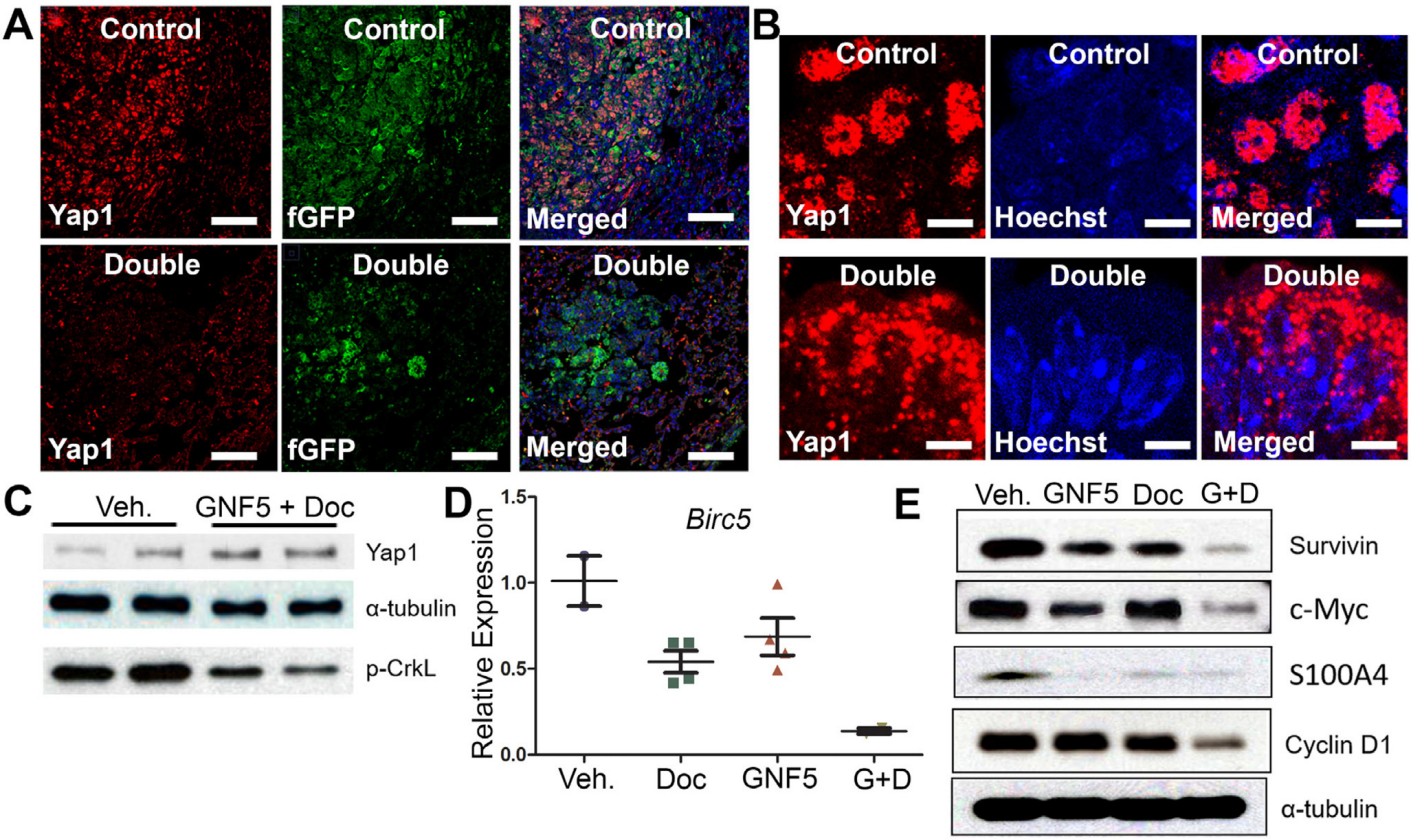
Combination treatment of GNF5 and docetaxel induces cytoplasmic localization of Yap1 and decreases expression of downstream transcription targets of Yap1 compared to vehicle control treated lung adenocarcinomas. GFP+ cells were isolated from *KRAS^G12D/+^; p53^-/-^; Rosa26-fGFP* mice two weeks after treatment with vehicle, docetaxel, GNF5, or combination treatment and 12 weeks after induction of tumors with adenovirus. (**A**) Immunofluorescence staining for Yap1 shows a decrease in Yap1 nuclear localization in mice treated with GNF5 and docetaxel compared to vehicle control treated mice. Scale = 75µm. (**B**) Higher magnification images are provided to show sub-cellular localization of Yap1 in control and combination treatment mice. Scale = 7.5µm. (**C**) Western blot (using the same lysates as Figure 2D) analysis showed no significant difference in total Yap1 protein expression in double treated mice compared to vehicle treated control mice. Phospho-CrkL (p-CrkL) expression is a marker of Abl kinase activity. (**D**) RT-qPCR analysis showed a decrease in mRNA transcript expression of the downstream Yap1 target, *Birc5*, which encodes the protein survivin, in mice treated with both GNF5 and docetaxel compared to vehicle control treated mice or mice treated with GNF5 or docetaxel alone (n= 3-4 mice per group, each RT-qPCR assay performed in triplicate). (**E**) Western blot analysis showed a decrease in protein expression of downstream transcriptional targets of Yap1, including survivin, c-Myc, S100A4, and cyclin D1. Graphs depict means and S.E.M.

