# Supplementary material for: ABL kinase inhibition sensitizes primary lung adenocarcinomas to chemotherapy by promoting tumor cell differentiation
Source: Oncotarget. 2019 Mar 8;10(20):1874–86. doi: 10.18632/oncotarget.26740 (PMC6443011; doi:10.18632/oncotarget.26740)
Supplement: Supplementary file 1 [file oncotarget-10-1874-s001.pdf]

ABL kinase inhibition sensitizes primary lung adenocarcinomas to chemotherapy by promoting tumor cell differentiation

SUPPLEMENTARY MATERIALS

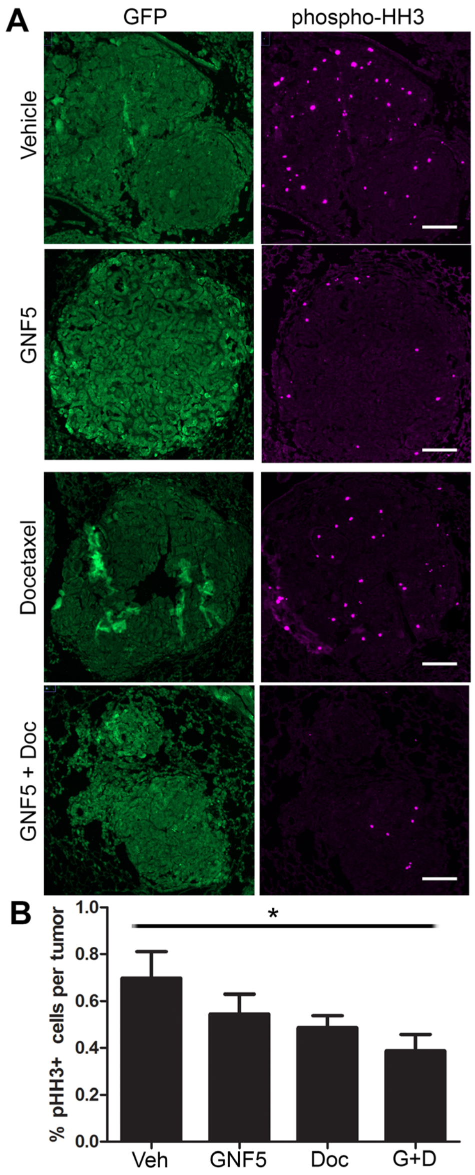

Supplementary Figure 1: Combination treatment of GNF5 and docetaxel significantly decreases expression of the cell mitosis marker, phospho-histone H3, in lung adenocarcinomas compared to vehicle control treated mice. Mice were treated with vehicle control, GNF5, docetaxel, or a combination of GNF5 and docetaxel 10 weeks after Adeno-Cre infection of *Rosa26-fGFP; LSL-Kras*$^{G12D/+}$*; p53*$^{fl/fl}$ mice. Mouse lungs were harvested at 12 weeks after Adeno-Cre infection. **A.** Representative immunofluorescence staining of tumor cells labeled with farnesylated GFP (left panels) and the mitotic cell marker pHH3 (right panels), showed a 50% decrease in the fraction of mitotic cells in lung adenocarcinomas in combination treatment mice compared to vehicle control treated mice. Scale bar = 100 µm. **B.** Quantification of immunofluorescence staining (n= 15-20 tumors from three individual mice per group). Graphs depict means and S.E.M.

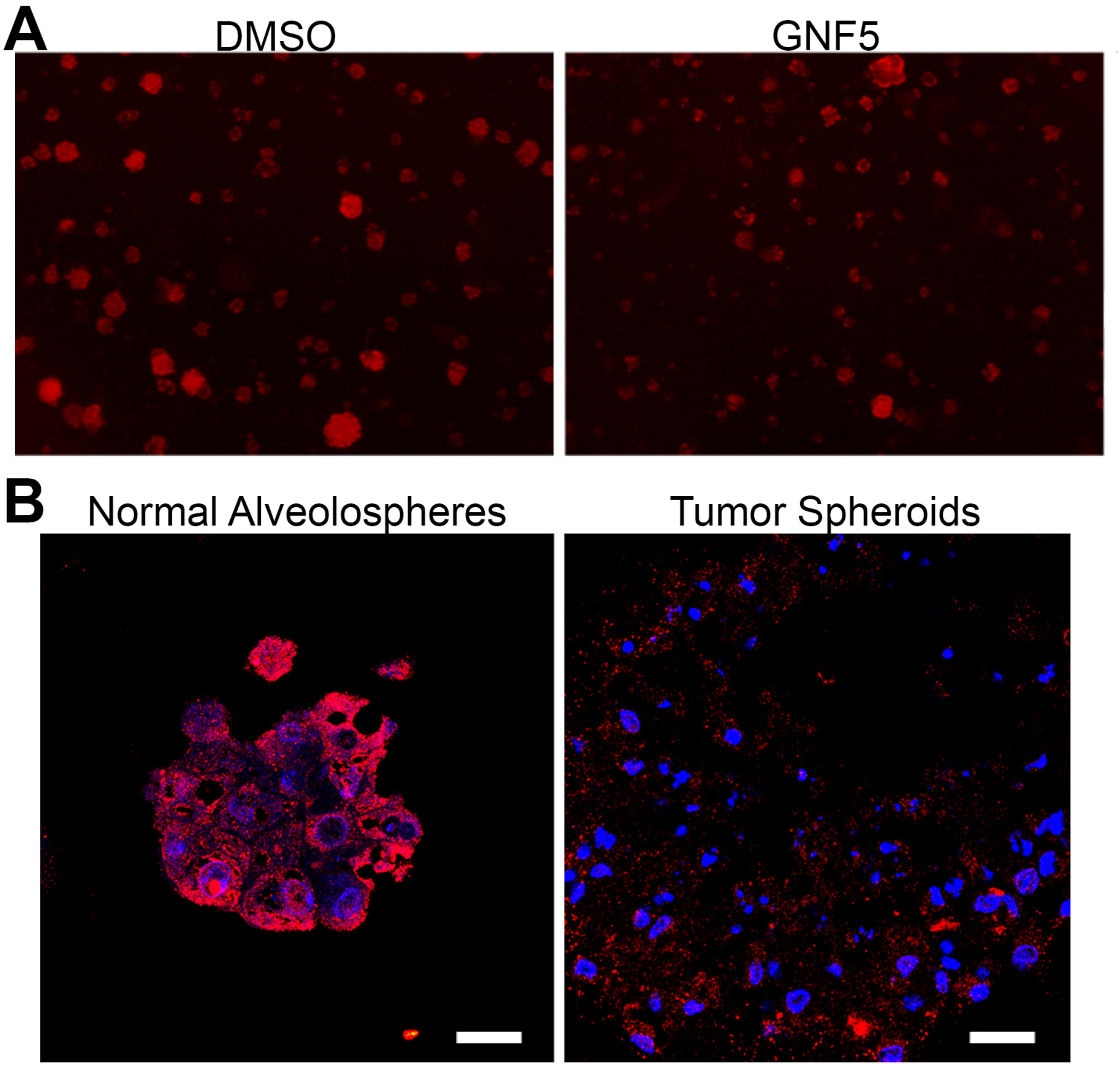

Supplementary Figure 2: Abl kinase inhibition does not affect normal alveolosphere growth and morphology; comparison of SPC expression in normal versus tumor spheres. A. *SPC (Sftpc)-CreERT2; Rosa26-tdTomato* wild type mice were given tamoxifen to induce tdTomato expression in Type II alveolar epithelial cells (AECs). Tomato+ cells were then isolated from lung tissue by FACS and grown in Matrigel in transwell inserts in the presence of primary mouse fibroblasts (derived from PDGFRα-H2B: GFP mice) to evaluate alveolosphere formation. Alveolospheres were treated with vehicle or GNF5 for 2 weeks and assessed for size and morphology. Treatment with the ABL kinase inhibitor, GNF5 (5 uM), does not significantly decrease 3D alveolosphere formation. **B.** Immunofluorescence staining for the Type II cell marker, SPC, shows a decrease in expression of SPC in organoids derived from KRAS-positive mice (tumor spheroids) treated with vehicle control, compared to organoids derived from KRAS-negative mice (normal lung alveolospheres). Scale bar = 10 µm.

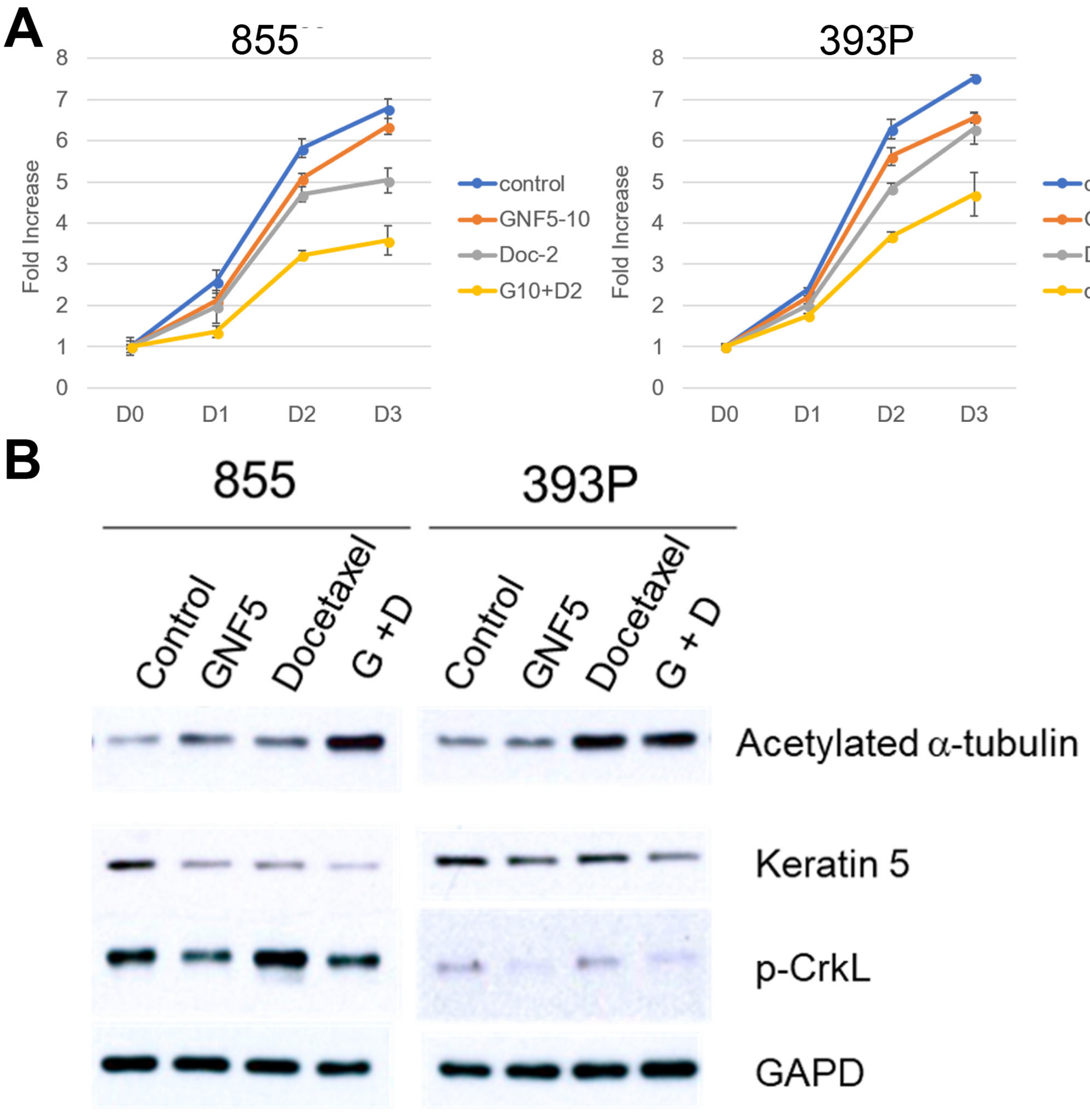

Supplementary Figure 3: Treatment with an ABL allosteric inhibitor sensitizes primary lung adenocarcinoma cells derived from *KRAS*$^{G12D+/-}$*; p53-/-* tumors to treatment with docetaxel and promotes tumor cell differentiation. A. CellTiter Glo was performed 24, 48, and 72 hours after plating two primary lung adenocarcinoma cell lines derived from *KRAS*$^{G12D+/-}$*; p53*$^{-/-}$ lung tumors. Treatment with a combination of GNF5 (10 µM) and docetaxel (2nM), resulted in a significant decrease in cell growth compared to treatment with either drug alone. **B.** Primary mouse lung adenocarcinoma cell lines derived from *KRAS*$^{G12D+/-}$*; p53*$^{-/-}$ tumors were treated with vehicle control, GNF5, docetaxel, or combination treatment for 48 hours. Western blot analysis of cell lysates showed an increase in expression of the ciliated cell marker, acetylated α-tubulin, with a corresponding decrease in expression of the basal cell marker, keratin 5, in mice treated with docetaxel and GNF5. Phospho-CrkL is a marker for ABL kinase activity; GAPD is a loading control.

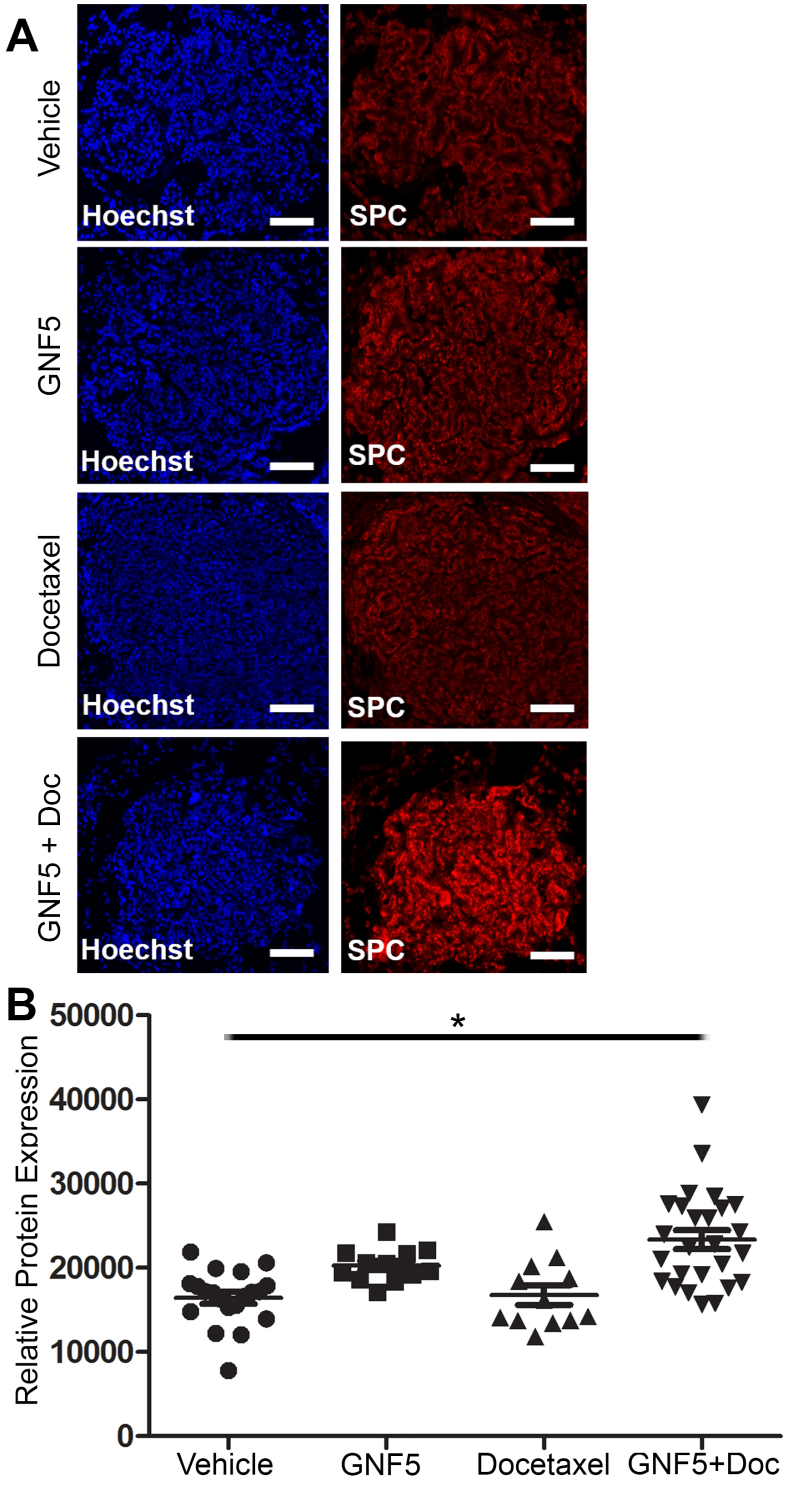

Supplementary Figure 4: Combination treatment of GNF5 and docetaxel significantly increases expression of the Type II alveolar epithelial cell marker, SPC in lung adenocarcinomas compared to vehicle control treated mice. Mice were treated with vehicle control, GNF5, docetaxel (Doc), or combination of GNF5 and docetaxel (G+D) 10 weeks after Adeno-Cre infection of *Rosa26-fGFP; LSL-Kras*$^{G12D/+}$*; p53*$^{fl/fl}$ mice. Mouse lungs were harvested at 12 weeks after Adeno-Cre infection. **A.** Representative immunofluorescence staining of tumor cells labeled with Hoechst33342 (left panels) and the type II alveolar epithelial cell marker, SPC (encoded by *Sftpc*) (right panels), shows an increase in SPC expression in lung adenocarcinomas in the mice treated with combination therapy compared to vehicle control treated mice. Scale bar = 100 µm. **B.** Quantification of relative protein expression per tumor area as measured by the integrated density function in the ImageJ software showed that combination treatment with GNF5 and docetaxel increased protein expression of the Type II cell marker, SPC, compared to vehicle control treated mice. Graphs depict means and S.E.M.
